# From hazard identification to risk assessment: The role of the prevention technician in the carcinogenic risk assessment for formaldehyde

**DOI:** 10.3389/fpubh.2023.960921

**Published:** 2023-04-14

**Authors:** Fabio Pattavina, Malgorzata Wachocka, Federica Tuti, Federica Boninti, Riccardo Santi, Roberta Grossi, Patrizia Laurenti

**Affiliations:** ^1^Hygiene Hospital Unit, Fondazione Policlinico Universitario A. Gemelli IRCCS, Rome, Italy; ^2^Ministry of Health, Rome, Italy; ^3^Life Sciences and Public Health Department, Università Cattolica del Sacro Cuore, Rome, Italy

**Keywords:** Cancerogen, formaldehide, risk assesment, hazard identification, algorithm

## Abstract

The Prevention Technician in the Environment and Workplaces (PTEW) is a health professional who works in the identification, assessment, and management of risk in living and working places. The PTEW implements specific corrective actions at reducing exposure levels to chemicals such as formaldehyde. The aim of this report was to update the formaldehyde risk assessment document (RAD). The risk assessment process was divided into three steps as follows: (1) preliminary data collection, (2) an on-site visit to identify the use patterns and process, and (3) application of the algorithm to calculate the exposure levels of healthcare workers. In addition, with the introduction of closed-circuit systems, 23 devices were evaluated to identify possible airborne dispersion of formaldehyde. The algorithm was applied in 31 hospital units and the results allowed us to classify the staff in two levels of exposure for each hospital unit; healthcare workers were classified as “exposed” or “potentially exposed.” Most of the HCWs are categorized as potentially exposed, and only workers working in laboratories are considered to be exposed. The results showed that devices must be used properly according to the user manual. To increase the level of worker safety, we have proposed to introduce closed-circuit safe handling systems and keeping the duration and intensity of exposure at the lowest possible levels according to the “ALARA” principle. The assignment of the Italian PTEW is to achieve excellence in the levels of health and safety of patients and hospital workers by pursuing a shared mission: improving the quality of public health.

## Introduction

The Prevention Technician in the Environment and Workplaces (PTEW) is a health professional who deals with the identification and characterization of risk in living and working places. One of the main tasks is to evaluate exposure to various substances, including carcinogens. The PTEW participates in different stages of risk management activities: inspections, hazard identification, risk assessment, environmental monitoring, and improvement proposals (1).

The risk assessment is the first among the general measures for the protection of the health and safety of workers. It is the tool used to guide and define preventive interventions (elimination, reduction, and/or control of risks), to plan information and training activities on risks and the protective measures adopted, and to observe the health status of workers (2). In addition, risk assessment is based on the acquisition of general theoretical scientific knowledge and carrying out field investigations (environmental and biological monitoring), leading to the estimation of the degree of exposure. Activities related to risk assessment include different aspects involving different professionals with distinct levels of responsibility (3).

The PTEW works in a team with different health professionals in departments of the territory that are essential for the identification of critical issues. Moreover, the PTEW identifies specific improvement measures aimed to reduce the concentrations of the dangerous substance (4, 5).

One of the agents for which an occupational exposure risk assessment is required is formaldehyde, which as of 2014 meets the criteria for classification as a carcinogen in Group 1B, according to EU Regulation N. 605/2014 (6). Internationally, as early as 15 July 2004, the International Agency for Research on Cancer (IARC) confirmed the carcinogenic effect of formaldehyde (7–10), before being classified only as a dangerous substance.

In Italy, for work activities involving exposure to formaldehyde, reference is made to Legislative Decree No. 81/2008—transposition of EU Directive 89/391/EEC (81/08) “protection from carcinogens and mutagens,” which defines the preventive actions to be implemented in workplaces in case of the use of carcinogens and/or mutagens.

The first recommendation is not to use and reduce the use of the substance and to replace it, if technically possible, with a chemical substance or mixture or process that is not harmful to health and safety.

If it is not technically possible, the carcinogens and/or mutagens must be replaced, ensuring that the production and/or use takes place in a closed system, and finally, if the previous measures are not feasible, the level of workers’ occupational exposure must be reduced to the lowest value (11, 12).

In Italy, a chemical frequently used in healthcare settings that requires a mandatory process of risk assessment is formaldehyde.

The use of formaldehyde in hospital settings is indispensable. However, technological advances offered by the healthcare technology and medical device market can enable the safe use of formaldehyde.

Although there are many studies highlighting how air ventilation, the use of “formaldehyde-free” chemicals, and continuous monitoring reduce occupational formaldehyde exposure, in recent years, closed-loop systems have been marketed of which a few studies assess formaldehyde dispersion (13–15).

The aim of this brief report is to analyze the methodological approach of workers’ carcinogenic risk management for the use of formaldehyde in healthcare work processes and closed-loop system dispersion assessment.

## Methods

The opportunity for this brief research report comes from the need to update the formaldehyde risk assessment document (RAD) in a University Hospital. The RAD is mandatory in Italy, according to Legislative Decree No. 81/2008.

### Formaldehyde risk assessment document

The first step of the risk assessment was data collection, i.e., a census was conducted to verify the hospital units where formaldehyde is used, and the annual quantity is used.

The second step was an on-site visit to identify formaldehyde as it was used and work processes. During this phase in hospital units, a checklist was applied. The information collected with the checklist was as follows:

- User’s professional role (physician, nurse, and technician).
- The number of people using the substance.
- Quantity of formaldehyde used in each process.
- Exposure time.

This information is essential to implement formaldehyde risk assessment and is used to estimate healthcare workers’ (HCWs) exposure through an algorithm, in compliance with the National Agencies for Environmental Protection ISPRA and ENEA (16).

In the third step, the algorithm was applied to calculate the exposure levels of healthcare workers (HCWs), according to the following formula:


$$\mathrm{Hcanc}\;\left(\mathrm{cancerogenic hazard}\right)=P\times CH\times T\times Q\times M\times F$$

where

- P: use and efficiency of PPE.
- CH: chemical/physical (gas, vapor, volatile liquid, and solid).
- T: temperature of the working process.
- Q: quantities used for each process.
- M: handling time (min/day).
- F: use frequency (days/year).

Table 1 shows that for each risk factor, a hazard score was attributed, depending on the risk category.

**Table 1:** Hazard score for each risk category.

Risk factor	Risk category		Hazard score
P	Closed loop and chemical hood		2
	Partially under chemical hood		5
	No chemical hood		10
CH	Gel,solid, compactṇ		2
	Non-volatile liquid, crystals		5
	Gas, volatile liquid vapor, fine dust		10
Q	<1 g	<1 ml	2
	1–50 g	1–50 ml	5
	>50 g	>50 ml	10
M	Minutes/Days		Minutes/480 (Working Minutes/Day)
F	Minutes/Years		Minutes/230 (Working Days/Year)

### Environmental sampling of closed-circuit security devices

During the period between November 2017 and October 2022, 23 closed-circuit systems were tested to assess the seal of the device to prevent any airborne leakage of formaldehyde. Table 2 shows the characteristics of the devices.

**Table 2:** Characteristics of the devices.

Id	Oevice capacity	Formaldeh yde	Buffer	Buffered formaldehyde	Batch	Expiration date
01		10 ml			00013	12/2018
02		20 ml			00004	06/2020
03		60 ml			00006	06/2020
04		900 ml			000020	01/2021
05		30 ml			1702	01/2022
06		60 ml			1729	07/2022
07		30 ml			201704	04/2019
08		90 ml			11601707	02/2019
09		10 ml			4117	10/2019
010		60 ml			00006	06/2020
011		20 ml			201711	11/2019
012		20 ml			201711	11/2019
013	60 ml	10 ml	10 ml		1806/6	06/2020
014	150 ml	60 ml		60 ml	1806/3	06/2020
015	60 ml	20 ml		20 ml	175/3	05/2019
016		110 ml	110 ml		1805/2	05/2022
017	40 ml			20 ml	00142	05/2022
018	60 ml	7 ml		33 ml	2022 × 00012	01/2024
019	60 ml	7 ml		33 ml	2022 × 00012	01/2024
020	250 ml	19 ml		110 ml	2022 × 31632	11/2022
021	90 ml				1130	03/2026
022	90 ml				1130	03/2026
023	90 ml				1130	03/2026

For airborne formaldehyde, measurements were used as a portable gas detector (RIKEN KEIKI HCHO Detector Mod. FP40). The measurement range is from 0.01 to 0.4 parts per million (ppm), with an interval of 0.01 ppm, and the value is expressed as <0.01 ppm, which was the detection limit of the instrument.

The standard measurement time was 3 min, after which the measurement results are readable on the instrument display.

The laboratory tests were carried out with the use of collective protective equipment (work under a chemical fume hood) and personal protective equipment (nitrile gloves as per the material safety data sheet indications of formaldehyde) to reduce any personnel exposure to a residual level.

The following measurement protocols were performed:

T1: device not used.
T2: immediately after device use.
T3: after 10 min after device use.
T4: after 1 h after device use.
T5: after 24 h after device use.

Before sampling, preparation of the instrumentation was carried out as follows:

- Cleaning the instrument measurement system, i.e., flushing the cell and internal duct by aspiration in air potentially formaldehyde free (outside air) for ~10 min.
- Cleaning of the system was repeated when the instrument measured the upper limit of the range (>0.4 ppm).
- In all other cases, several cycles of “refresh,” i.e., vacuum cleaning of the system, were performed between measurements.
- Measurements were made inside a glass container with a volume of ~10 L equipped with an air inlet valve.

## Results

The algorithm was applied in 31 hospital units, and the results allowed us to classify the staff into two levels of exposure for each hospital unit; the HCWs were classified as “exposed” or “potentially exposed.”

Table 3 shows the results of the evaluation by individual healthcare professional category.

**Table 3:** Results HCWs potentially exposed or exposed in hospital unit.

Hospital units *N* = 31	HCWs potentially exposed in HU *n* (%)	HCWs exposed in HU *n* (%)
Doctor	25 (80.6)	2 (6.4)
Nurse	21 (67.7)	2 (6.4)
Midwife	3 (14.2)	0 (0.0)
Technician	1 (4.8)	3 (9.7)

HU: hospital unit.

Most of the healthcare categories result as potentially exposed, the hospital units with the doctor category potentially exposed were 25 (80.6%), and the hospital units with the nurse category potentially exposed were 21 (67.7%); however, the hospital units with the technician category potentially exposed were 3 (9.7%).

Table 4 shows the results of the environmental samplings to assess the seal of the device.

**Table 4:** Environmentlsamplings of the closed-circuit devices.

ID	T1 (ppm) (PPM)	T2 (ppm)	T3 (ppm)	T4 (ppm)	T5 (ppm)
1	<0.01	<0.01	<0.01	0.02	0.04
2	<0.01	<0.01	<0.01	0.03	0.09
3	0.01	0.01	<0.01	<0.01	0.14
4	0.01	0.01	<0.01	<0.01	0.07
5	0.01	0.07	0.1	0.26	**>0.4**
6	<0.01	0.05	0.07	0.21	**>0.4**
7	<0.01	<0.01	<0.01	<0.01	0.01
8	<0.01	0.01	0.01	0.06	**>0.4**
9	<0.01	0.09	**>0.4**	**>0.4**	**>0.4**
10	0.07	0.02	0.06	0.1	0.28
11	<0.01	0.22	**>0.4**	**>0.4**	**>0.4**
12	<0.01	0.31	**>0.4**	**>0.4**	**>0.4**
13	0.05	0.06	0.07	0.13	0.25
14	0.12	0.12	0.12	0.12	**>0.4**
15	<0.01	0.03	0.1	0.15	**>0.4**
16	<0.01	<0.01	<0.01	0.06	0.06
17	**>0.4**	**>0.4**	**>0.4**	**>0.4**	**>0.4**
18	0.01	**>0.4**	**>0.4**	**>0.4**	**>0.4**
19	<0.01	0.05	0.06	0.06	0.04
20	0.01	<0.01	<0.01	0.25	0.18
21	0.02	<0.01	0.01	<0.01	<0.01
22	0.01	<0.01	0.01	<0.01	0.02
23	0.02	0.01	0.01	<0.01	0.03

Bold value indicates are measured values but out of the measurement scale.

## Discussion

In relation to the results, corrective actions were implemented, to ensure workers’ health and safety levels over time:

1. **Closed-circuit safety device** was introduced for the safe handling of small histological biopsy.
2. **Purchase of closed-circuit safety equipment** for handling jugs with formaldehyde. In hospital units, where is not possible to use the closed-circuit safety jug because of the handling of large anatomical pieces, the risk of exposure for healthcare professionals remains higher.
3. **Environmental sampling**: In total, 23 different types of closed-circuit safety were tested to verify the seal of the device. Furthermore, three closed-circuit safety equipment for the automatic filling of large containers were evaluated to verify the level of dispersion of formaldehyde vapors during their use. The data show that if the device is used correctly, the levels of environmental contamination are to be considered harmless; on the contrary, if the device is not used correctly, the contamination levels exceed the measurement range.
4. **Accidental spill containment kit**: A kit has been prepared for use in the event of an accidental formaldehyde spill. The kit consists of the necessary personal protective equipment (PPE), neutralizing cloths, a bag, and a bucket for disposal. In addition, training was conducted on the proper use of the emergency kit. These kits have been stocked in all hospital units where it is necessary to store formaldehyde containers.
5. **Updating of the accidental spill procedure**: The internal procedure was updated with a description of the new operating instructions to be adopted in the case of accidental spillage of small quantities (≥10 ml to ≤10 L) of hazardous substances or chemicals. The new internal procedure was implemented to reduce the risk and define the area to be isolated and cleaned, according to the new material safety data sheet.
6. **Training**: The training was planned for work classified as “exposed” and organized into three courses each lasting 2 h. The total number of workers who participated was 75. Topics were an update on the appropriate use of formaldehyde in accordance with the internal procedure, the proper use of new closed-circuit safety devices, PPE to be used, and what to do in case of an accidental spill.

## Conclusion

The results showed that devices must be used properly, according to the user manual, to avoid any contamination.

The application of a risk assessment methodology is critical to evaluate healthcare professionals’ exposure to formaldehyde. This methodology estimates the efficacy of a series of protective actions for the health and safety of workers and how to better manage the risk.

To increase the level of worker safety, we have proposed to introduce closed-loop or safe handling systems and keeping the duration and intensity of exposure at the lowest possible levels, according to the “as low as reasonably achievable” (ALARA) principle (17).

The formaldehyde risk assessment and management are considered a priority for the health and safety of HCWs and involve a multidisciplinary group to develop a method of updating the risk assessment considering international and national laws and guidelines.

The task of the Italian Prevention Technician is to achieve excellence in the levels of health and safety of patients and hospital workers by pursuing a shared mission: improving the quality of public health.

## Data availability statement

The original contributions presented in the study are included in the article/supplementary material, further inquiries can be directed to the corresponding author.

## Author contributions

FP, FT, MW, FB, RG, and RS had the idea and contributed to the writing of the text. PL carried out the final revision. All authors contributed to the article and approved the submitted version.

## Conflict of interest

The authors declare that the research was conducted in the absence of any commercial or financial relationships that could be construed as a potential conflict of interest.

## Publisher’s note

All claims expressed in this article are solely those of the authors and do not necessarily represent those of their affiliated organizations, or those of the publisher, the editors and the reviewers. Any product that may be evaluated in this article, or claim that may be made by its manufacturer, is not guaranteed or endorsed by the publisher.
